# The carbon intensity (CI) of co-products fed to beef cattle in the Northern Plains

**DOI:** 10.1093/tas/txag137

**Published:** 2026-09-18

**Authors:** Felipe Almeida, Richard K Perrin, Lilyan E Fulginiti, Elliott Dennis

**Affiliations:** Department of Agricultural Economics, University of Nebraska, Lincoln, NE 68583, United States; Department of Agricultural Economics, University of Nebraska, Lincoln, NE 68583, United States; Department of Agricultural Economics, University of Nebraska, Lincoln, NE 68583, United States; Department of Agricultural Economics, University of Nebraska, Lincoln, NE 68583, United States

**Keywords:** carbon intensity (CI), beef production, life cycle assessment, distillers grains, soybean meal, Northern Plains

## Abstract

The beef production chain accounts for about 40% of world livestock greenhouse gas (GHG) emissions, with feed production representing a substantial component of livestock’s carbon footprint. Two important components of livestock rations in the United States are co-products from grain processing: soybean meal (SBM) from soybean crushing, and distillers grains and solubles (DGS) from corn ethanol production. In response to interest in low-carbon beef production, this study estimates the likely carbon intensity (CI) scores of these two co-product feeds, using two life-cycle emissions packages available for use by producers and others. For feedstuffs produced in the Northern Plains we find expected CI estimates of 0.196–0.385 kg CO₂e kg^−1^ of dry matter for DGS and 0.330–0.456 kg CO₂e kg^−1^ of dry matter for SBM. The analysis reveals that expected co-product CIs for a given product and model vary by 15%–19% across subregions in which the crop was grown, and that CI of co-products from irrigated crops in this region are about 10% lower than those from non-irrigated crops. These results suggest that beef producers may find opportunities to reduce their product CI by choosing among available supplies of distillers grains and soybean meal.

## Teaser

Beef producers in the Northern Plains have the potential to choose co-product feedstuffs with a range of carbon intensity (CI), measured here as kg of CO_2_e kg^−1^ of feed dry matter. CIs for dried distillers grains and solubles range from 0.196 to 0.385, while for soybean meal the range is from 0.330 to 0.456, depending on the subregion, irrigation status and the model used to estimate crop CI.

## Introduction

The beef production chain is responsible for about 40% of global livestock greenhouse gas emissions (GHG) (Gerber et al. 2013). In the United States this has led to interest in the potential for more sustainable beef production, both by researchers (Rotz et al. 2013) and by producers (https://attra.ncat.org/climate-solutions/). Because harvested feedstuffs constitute a significant proportion of the carbon footprint of US beef production (Rotzet al. 2019), lower-carbon feedstuffs may provide one avenue for improving the sustainability of beef production. This study examines carbon emissions intensities (CI) of important co-products fed to beef, measured as kg CO_2_e kg^−1^ feedstuff dry matter (DM).

Two important ingredients in beef rations are distillers grains and solubles (DGS) and soybean meal (SBM). Currently, beef producers in the Northern Plains do not know the CI of the co-products they are feeding. A recent meta-analysis (Springer et al. 2026) found six publications from 2012 to 2024 with estimates of the CI of US soybean meal, ranging from 0.441 to 1.178 kg CO_2_e kg^−1^. We have found only two life-cycle assessment (LCA) estimates of the CI for DGS, those being 0.242 kg CO_2_e kg^−1^ (Haque et al. 2022, as fed basis) and 0.910 (Adom et al. 2012, DM basis). While there are a number of estimates of the CI of US SBM, they vary considerably by region, production practices and measurement boundaries. Here we examine the CI of these feedstuffs that producers in the Northern Plains should expect to encounter (measured in this paper as kg of carbon dioxide equivalent [CO_2_e] per kg of feedstuff DM), and how that CI might vary within the region and with production practices.

The objectives of this study are therefore threefold: (i) to estimate the expected CI of DGS (as wet distillers grains with solubles [WDGS], modified distillers grains with solubles [MDGS], and dried distillers grains with solubles [DDGS]) and of soybean meal (SBM) produced in the Northern Plains, using a transparent value-based allocation of emissions among co-products; (ii) to quantify how these co-product CIs vary across subregions and between irrigated and non-irrigated production; and (iii) to compare estimates derived from two publicly available life cycle models (GREET and IFSM) so that producers and others can judge the range of plausible values for their own feedstuffs.

## Materials and methods

### Soybean meal (SBM) and distillers grains and solubles (DGS)

We examine the CI of SBM and DGS produced in the Northern Plains. SBM is co-produced with soybean oil in soybean processing (This process begins with the cleaning and thermal conditioning of soybeans, preparing them for dehulling and subsequent flaking. The crucial step is oil extraction, primarily conducted using organic solvents such as hexane. The residual solid material, after oil separation and solvent removal through evaporation, forms the meal. To enhance its nutritional value and remove thermolabile antinutritional factors, the meal undergoes a final heat treatment (Dalgaard et al. 2008; Banaszkiewicz 2011; Dei 2011; Benavides et al. 2020). SBM has a high dry matter (DM) content of around 87-90% and a crude protein of 44-49% (on a DM basis) (Banaszkiewicz 2011; Mukherjee et al. 2016).). DGS is co-produced with ethanol and other co-products, primarily from corn grain processing using the dry mill process (The dry mill process begins with the milling of the corn. The corn is then mixed with water and enzymes to produce mash that is subsequently fermented by yeasts that convert the starch into ethanol. After fermentation, the mixture is distilled to separate the ethanol, leaving behind stillage composed of unfermented solids, proteins, fibers, fats, and other nutrients.). In the dry mill process the stillage remaining after distillation is used to produce various forms of DGS, each of which we examine: wet distillers grains with solubles (WDGS) which has a high moisture content (60%–80%); modified distillers grains with solubles (MDGS) with intermediate moisture content (around 50%); and dry distillers grain with solubles (DDGS) dried to around 11% moisture (DDGS is widely used in animal nutrition, providing protein, fat, and fiber, and serving as an excellent energy source (Belyea et al. 2004; Mumm et al. 2014; Dennis et al. 2024). Despite their moisture differences, all three distillers’ co-products generally provide a robust crude protein content of 28%–35% (on a DM basis) (Hoffman and Baker 2010; Liu 2011; Mohammadi Shad et al. 2021).).

Carbon emissions attributable to these co-products consist of emissions attributable to grain production and emissions attributable to processing. For estimates of corn and soybean production emissions grown in this region, we use results from Perrin et al. (2026). We then use the market value share approach to allocate grain production emissions among the by-products.

### Emissions attributable to feedstock production

The feedstock component of co-product CI can be calculated from the CI of the grain. For the CI of grain produced in this region, we use the Perrin et al. (2026) life cycle assessment (LCA) estimates for corn and soybean CIs, which do not include any emissions due to indirect land use change nor emissions due to change in soil organic matter. That study derived estimates using two alternative life-cycle assessment (LCA) models; the Greenhouse gases, Regulated Emissions, and Energy use in Technologies (GREET), an inventory-based model maintained by the US Department of Energy; and the Integrated Farm Simulation Model (IFSM), a day-step crop growth model developed and maintained by the US Department of Agriculture (GREET is available for public use from https://www.energy.gov/cmei/greet, IFSM is available for public use at https://www.ars.usda.gov/northeast-area/up-pa/pswmru/docs/integrated-farm-system-model/.). The results they report by crop, irrigation status and subregion are those in Table 1. The weighted average CIs for corn (CI_corn_) are 0.252 and 0.290 kg CO₂e kg⁻¹ of grain DM for the GREET and IFSM models, respectively. The weighted average CIs for soybean (CI_soybean_) are 0.321 and 0.284 kg CO₂e kg⁻¹ of grain DM for the GREET and IFSM models, respectively.

**Table 1:** Expected life-cycle carbon intensity (CI) scores for production of corn and soybeans in the Northern plains under standard production practices (no soil organic carbon or indirect land use emissions included).

Region	Corn		Soybeans	
	IFSM	GREET	IFSM	GREET
	kg CO_2_e kg^−1^ of grain DM			
**East Nebraska**				
** Non-irrigated**	0.320	0.256	0.310	0.329
** Irrigated**	0.280	0.242	0.270	0.307
**Central Nebraska**				
** Non-irrigated**	0.280	0.311	0.300	0.418
** Irrigated**	0.270	0.245	0.250	0.307
**West Nebraska**				
** Non-irrigated**	0.370	0.335	NA	NA
** Irrigated**	0.280	0.252	0.340	0.329
**Nebraska averages^a^**				
** Non-irrigated**	0.314	0.267	0.309	0.336
** Irrigated**	0.276	0.244	0.263	0.307
** weighted average**	0.290	0.252	0.284	0.321

a Using recent NASS production share weights; NA, not applicable.

Source: Perrin et al. (2026).

We use the market value share to allocate the emissions from grain production and processing emissions among the co-products of processing (Guinée et al. 2004; Wang et al. 2011; Liu 2019; Benavides et al. 2020). For corn ethanol processing, we use the average 2019–2023 shares of ethanol plant co-products (Iowa State University, https://www.extension.iastate.edu/agdm/energy.html, accessed July 27, 2025.) which were DGS 22%, ethanol 74% and corn oil 4%. For soybean processing, we use the recent 5-year value shares for soybean processing plant co-products which were 40% (rounded down from 40.7%) for soybean oil and 60% (rounded up from 59.3%) for SBM with hulls (Oil Crops Yearbook [Economic Research Service (ERS), 2025], www.ers.usda.gov/data-products/oil-crops-yearbook, accessed July 27, 2025.).

The portion of the co-product CI attributable to the production of the grain feedstock can be calculated from the CI of the grain. For DGS, 0.33 kg of DGS DM is extracted from a kg of corn, so considering that 22% of feedstock emissions are allocated to DGS, the feedstock component of the DGS CI is calculated as:


(1) feedstock CIDGS=0.22CIcorn0.33=0.667(CIcorn) 

For SBM, 19.02 kg of SBM DM is produced from the 23.68 kg of DM in a bushel of soybean, or the amount of soybean required to produce a kg of SBM DM is 23.68/19.02 = 1.245 kg soybean DM kg^−1^ SBM DM. Total emissions in that amount of soybean is thus 1.245*CI_soybean_. We allocate 60% of these emissions to SBM, thus calculating the portion of SBM emissions from soybean production as:


(2) feedstock CISBM=0.60(1.245)CIsoybean =0.75(CIsoybean)

### Emissions attributable to processing

For corn processed in dry mill ethanol plants, basic processing produces WDGS with about 80% moisture, which can then be dried further to produce MDGS (about 50% moisture) or DDGS (11% moisture). Emissions from the basic processing stage are allocated using the value share for DGS above, 22%. We estimate these basic processing emissions at 0.0345 kg CO_2_e kg^−1^ of DM in DGS, as follows. Scully et al. (2021) determined the central best estimate of emissions during ethanol processing to be 29.6 g CO_2_e MJ^−1^, or 0.165 kg CO_2_e L^−1^. Gallagher et al. (2016) (Using data from Table 2 in Gallagher et al. (2016).), determined that 0.0657 kg of this 0.165 is due to drying to produce DDGS, leaving 0.0993 kg due to basic processing that produces WDGS. At 0.6315 kg DGS DM L^−1^, this amounts to (0.165 − 0.0657)/0.6315 = (0.0993)/0.6315 = 0.157 kg CO_2_e kg^−1^ of DM in WDGS due to basic ethanol processing, of which we allocate 22% to WDGS, or 0.22(0.157) = 0.0345 kg CO_2_e kg^−1^ of DM.

**Table 2:** Life-cycle carbon intensity (CI) benchmarks for distillers grains with solubles (DGS) and soybean meal (SBM) from crops produced in the Northern plains (no soil organic carbon or indirect land use emissions included).

	WDGS		MDGS		DDGS		SBM	
	IFSM	GREET	IFSM	GREET	IFSM	GREET	IFSM	GREET
	kg CO_2_e kg^−1^ of co-product DM							
**East Nebraska**								
** Non-irrigated**	0.248	0.205	0.298	0.255	0.352	0.309	0.375	0.389
** Irrigated**	0.221	0.196	0.271	0.246	0.325	0.300	0.345	0.372
**Central Nebraska**								
** Non-irrigated**	0.221	0.242	0.271	0.292	0.325	0.346	0.367	0.456
** Irrigated**	0.215	0.198	0.265	0.248	0.319	0.302	0.330	0.372
**West Nebraska**								
** Non-irrigated**	0.281	0.258	0.331	0.308	0.385	0.362	NA	NA
** Irrigated**	0.221	0.203	0.271	0.253	0.325	0.307	0.397	0.389
**Nebraska averages^a^**								
** Non-irrigated**	0.244	0.213	0.294	0.263	0.348	0.317	0.374	0.394
** Irrigated**	0.219	0.197	0.269	0.247	0.323	0.301	0.339	0.372
** Weighted average**	0.228	0.203	0.278	0.253	0.332	0.307	0.355	0.383

DM, dry matter; DGS, distillers grains and solubles; WDGS, wet distillers grains and solubles; MDGS, modified distillers grains and solubles; DDGS, dried distillers grains and solubles; SBM, soybean meal; IFSM, Integrated Farm Systems Model; GREET, Greenhouse Gases, Regulated Emissions, and Energy Use in Technologies model; NA, not applicable.

a Using recent NASS production share weights.

For MDGS and DDGS, the 0.0657 kg CO_2_e L^−1^ due to drying must be allocated to the dried products. DDGS requires the entire drying process for which the emissions amount to 0.0657/0.6315 = 0.104 kg CO_2_e kg^−1^ DM of DDGS. We have no data that allows us to adjust the drying emissions for MDGS relative to DDGS, so we arbitrarily assume the drying emissions for MDGS to be one-half that of DDGS or 0.104/2 = 0.052 kg CO_2_e kg^−1^ DM of MDGS.

For soybean processing, we use the USB-NOPA estimate of emissions, 0.126 kg CO_2_e kg^−1^ of crude soybean meal (United Soybean Board (USB) and National Oilseed Processors Association (NOPA) (2024).). At 11% DM, the processing emissions are thus 0.126(0.89)^−1^ = 0.142 kg CO_2_e kg^−1^ of DM.

Summarizing these calculations, co-product CIs are comprised of a component derived from production of the grain feedstock plus a component due to processing, as follows:


(3) CIWDGS=0.667CIcorn+0.0345

(4) CIMDGS=0.667CIcorn+0.0345+0.052 

(5) CIDDGS=0.667CIcorn+0.0345+0.104 

(6) $$CI_{\mathrm{SBM}}=0.75CI_{\mathrm{soybean}\;}+0.142$$

## Results

In Table 2 we present the results from using Equations (1)–(6) to estimate co-product CIs (all measured as kg CO_2_e kg^−1^ of co-product DM) from the feedstock grains in Table 1. The estimates of WDGS CI range from 0.196 to 0.281 kg CO_2_e kg^−1^ of co-product DM. Given the GREET estimates of corn feedstock emissions, WDGS CI estimates range from 0.196 to 0.258 kg CO_2_e kg^−1^ of co-product DM, for a weighted average of 0.203, about 11% lower than the comparable IFSM-based weighted average of 0.228. Due to emissions from drying, MDGS has an average estimated CI about 23% higher than WDGS, while DDGS is 48% higher, at 0.307–0.332 kg CO_2_e kg^−1^ of co-product DM.

CI for soybean meal, ${CI}_{\mathrm{SBM}}$, evaluated at the IFSM CI estimates for soybean production from Table 1, ranges from 0.330 to 0.375, or a weighted average of 0.355. Comparable estimates based on GREET CI estimates are from 0.372 to 0.456, or a weighted average of 0.383.

We found that in this region estimates of co-product CIs from irrigated crops are about 10% lower than those from rainfed crops. Co-product CIs from crops across subregions differ by 15%–23% under non-irrigated production, 2%–18% under irrigation. Estimated CIs for MDGS are roughly 25% higher than those for WDGS, while CIs for DDGS are roughly 50% higher, due to drying emissions.

## Discussion

The CIs of feedstuffs are an important component for evaluating the carbon footprint of beef and other livestock products. This research examines the likely CI scores (kg CO_2_e kg^−1^ DM) that livestock producers in this region might encounter for two such feedstuffs, DGS and SBM. At the present time, the burden of establishing the CI of individual batches of feedstuffs would be the responsibility of the beef producer. However, this is changing soon with the June 25, 2026, USDA release of the Regenerative Feedstock Rule that establishes the Feedstock Carbon Intensity Calculator for field-level GHG calculations (See https://www.usda.gov/technical-guidelines-regenerative-biofuel-feedstocks.). While that rule establishes how corn and soybean CI is to be calculated for purposes of the clean fuel (“45Z”) program, the rule will make it feasible for crop producers to establish their crop CI for any marketing purpose. Hence it is likely that beef producers may soon have market access to co-products with verifiable feedstock CIs. The results of the present research will provide background for evaluating those alternatives.

Springer et al. (2026) found six publications from 2012 to 2024 with estimates of the CI of US SBM, ranging from 0.441 to 1.178 kg CO_2_e kg^−1^, far higher than our estimates. Benavides et al. (2020) estimate the US-wide SBM CI at 0.400 using market allocation, 0.482 using mass allocation, still somewhat higher than our estimates. Benavides et al. (2020) also estimate US-wide DDGS CI at 0.558 kg CO_2_e per kg of product (not per kg of product DM) using value allocation (0.869 with mass allocation), much higher than our estimates. Hence producers in the Northern Plains region may have a slight advantage in producing lower-CI beef because of lower co-product CIs.

Regarding the method of allocating emissions among co-products, the International Organization for Standardization (ISO) (2006) proposes a hierarchy of approaches: (i) subdividing multifunctional processes into mono-functional processes with distinct inputs and outputs such as Ekvall and Finnveden (2001), which is not possible for co-products that are produced jointly; (ii) system expansion such as Kim and Dale (2002), Cederberg and Stadig (2003), Suh et al. (2010), Uusitalo et al. (2014), and (iii) allocation based on some relevant property of the co-products, such as share of mass or share of market value such as Guinée et al. (2004), Uusitalo et al. (2014) and Michiels et al. (2021). We argue that market value/economic allocation is the most logical approach because it results in the carbon emissions burden for co-products being proportional to the human benefits each brings to society, as reflected by their market values. Furthermore, these relative values reflect the relative intensity of why the co-products were produced at all. Any co-products that do not have positive market value are normally considered “wastes” and should not share any of the carbon emissions burden. We note that the choice of allocation factor can matter considerably. But even mass allocation is not definitive: calculated allocation for DGS based on relative mass allocation has ranged from 35% to 53%. Based on relative economic values the same allocation ranges from 21% to 33% (see Table A1 in Supplementary Information), compared to the 22% that we use. Published SBM mass allocation factors have ranged from 71% to 80%, compared to the 40% value share we use. It is true that value shares change through time (see Table B2, Supplementary Information), but it is nonetheless an appropriate allocation to use, as co-products have a greater or smaller importance to the economy through time.

It is important to note that boundaries can matter considerably to life-cycle emissions analysis. Our study does not include emissions due to indirect land use change, which is appropriate because they are not included in the USDA Regenerative Feedstock Rule. Neither have we included emissions due to changes in soil organic matter because science has not settled upon generalized quantitative measurements. The new USDA FD-CIC tool does, however, include estimates of emission changes due to changes in soil organic matter, so results obtained with that new tool may differ somewhat from those obtained here based on GREET and IFSM.

The ranges of CIs we found are relatively modest in absolute terms but may be somewhat more meaningful for a producer who is working to formulate a low-carbon ration. If harvested feeds account for roughly 13% of the carbon footprint of US beef production (Rotzet al. 2019), a 10% reduction in the CI of a co-product feed would translates into only about a 1% change in whole-system beef CI when that feed is one component of the diet. We believe that the low-carbon issue is potentially more significant in the choice of the form of distiller grains to use because drying accounts for the entire difference between the CIs of WDGS and DDGS. A producer with access to wet or modified distillers grains from a nearby ethanol plant could avoid the drying emissions that would raise DDGS CI to roughly 50% more than WDGS. Proximity to an ethanol plant, which makes wet co-products economical to transport, is therefore just as relevant to the feed/ration CI as the subregion or irrigation status of the underlying crop. Where producers source from several supply points, the subregional and irrigation differences which we document in Table 2 provide a basis for preferring lower-CI supplies when price and availability allow.

We condition our interpretation of the results with several limitations. First, value-based allocation ties the emissions burden to relative market prices, but market prices need not reflect the social value where products are subsidized or where externalities are unpriced. Ethanol markets in particular are shaped by policy, so the value shares used here embed those policy conditions. We nonetheless prefer value allocation because it assigns burden in proportion to the economic reason the co-products are produced, and because it is the only standard method that consistently excludes zero- or negative-value residues. Second, the drying emissions for MDGS we have assumed to be half those of DDGS in the absence of process data specific to the modified product. Because the moisture reduction in producing MDGS (from roughly 65% to 50%) is smaller than that removed for DDGS (to roughly 11%), we believe that this assumption is plausible though approximate, and the MDGS column should be read as indicative. Third, all crop CIs are taken from Perrin et al. (2026) and exclude emissions from change in soil organic carbon and those from indirect land use change, consistent with the new USDA Regenerative Feedstock Rule.

## Conclusions

Using a transparent value-based allocation of emissions among co-products, we estimate expected carbon intensities of 0.196–0.385 kg CO_2_e kg^−1^ of DM for distillers grains and 0.330–0.456 for SBM produced in the Northern Plains. These values vary by subregion, by irrigation status, and, for distillers grains, by the degree of drying, with the drying difference between wet and dried distillers grains being the single largest source of variation. The estimates are lower than most previously published figures for these feeds, reflecting both the regional crop CIs and the value-based allocation. They give producers and others a defensible basis for comparing the carbon intensity of available co-product feeds and for identifying lower-carbon supplies where price and availability permit.

## Supplementary Material

txag137_Supplementary_Data

## Abbreviations

CI: carbon intensity
CO_2_e: carbon dioxide equivalent
DGS: distillers grains and solubles
DDGS: dried distillers grains and solubles
DM: dry matter
GHG: greenhouse gas
GREET: Greenhouse Gases, Regulated Emissions, and Energy Use in Technologies
IFSM: Integrated Farm Systems Model
ISO: International Organization for Standardization
LCA: life cycle assessment
MDGS: modified distillers grains and solubles
SBM: soybean meal
WDGS: wet distillers grains and solubles
