# Supplementary material for: The carbon intensity (CI) of co-products fed to beef cattle in the Northern Plains
Source: Transl Anim Sci. 2026 Sep 18;10:txag137. doi: 10.1093/tas/txag137 (PMC13623517; doi:10.1093/tas/txag137)
Supplement: Supplementary file 1 [file txag137_supplementary_data.zip › 14-Sep-2026_100519_SUPPLEMENTAL_MATERIALSr3_clean.docx]

**SUPPLEMENTAL INFORMATION**

**A: Studies Reporting CI Allocation by Mass, Economic Value, and Energy**

In this research, we use the economic value method for allocating emissions among co-products. Other allocations for DGS and SBM that we have found in the literature are reported in Table A1.

**Table A1. DGS and SBM allocations**

| **Source** | **Coproduct** | **Mass Allocation** | **Economic Value Allocation** | **Energy Allocation** |
| --- | --- | --- | --- | --- |
| Wang et al. (2011) | DGS | 44% | 22% | 37% |
| Wang et al. (2012) | DGS | 35%* |  |  |
| Liu (2019) | DDGS | 51% | 33% |  |
| Benavides et al. (2020) | DDGS | 46% | 22% |  |
| Wu et al. (2024) | DDGS | 53% | 21% |  |
| Hou et al. (2009) | SBM |  | 52.6-70.1% | 55.3-75.9% |
| Omni Tech International (2010) | SBM | 80.5% | 57-70% |  |
| Milazzo et al. (2013) | SBM |  | 64% |  |
| Reckmann et al. (2013) | SBM |  | 66.3% |  |
| Han et al. (2014) | SBM | 79% |  |  |
| Chen et al. (2018) | SBM |  |  | 30.3% |
| Liu (2019) | SBM |  | 63% |  |
| Benavides et al. (2020) | SBM | 78% | 65% |  |
| Xu et al. (2022) | SBM | 78.4% | 68.1% | 56.6% |
| Wu et al. (2024) | SBM | 71% | 56% |  |

Note: *on a dry matter basis.

**B. Revenue shares for DGS and SBM over time.**

**Figure B1. Average revenue shares of DGS in ethanol processing products, 2016-2025^a^**

|  | #REF! | monthly | 5-yr moving avg |
| --- | --- | --- | --- |
| 2005-01-01 | 1 |  |  |
| 2016-01-01 |  | 0.213512 |  |
| 2016-02-01 |  | 0.205723 |  |
| 2016-03-01 |  | 0.210665 |  |
| 2016-04-01 |  | 0.183217 |  |
| 2016-05-01 |  | 0.198321 |  |
| 2016-06-01 |  | 0.221273 |  |
| 2016-07-01 |  | 0.207633 |  |
| 2016-08-01 |  | 0.196033 |  |
| 2016-09-01 |  | 0.177412 |  |
| 2016-10-01 |  | 0.15775 |  |
| 2016-11-01 |  | 0.154261 |  |
| 2016-12-01 |  | 0.143332 |  |
| 2017-01-01 |  | 0.160279 |  |
| 2017-02-01 |  | 0.162283 |  |
| 2017-03-01 |  | 0.160283 |  |
| 2017-04-01 |  | 0.143487 |  |
| 2017-05-01 |  | 0.150954 |  |
| 2017-06-01 |  | 0.153388 |  |
| 2017-07-01 |  | 0.162838 |  |
| 2017-08-01 |  | 0.167841 |  |
| 2017-09-01 |  | 0.159727 |  |
| 2017-10-01 |  | 0.176186 |  |
| 2017-11-01 |  | 0.190783 |  |
| 2017-12-01 |  | 0.214507 |  |
| 2018-01-01 |  | 0.227126 |  |
| 2018-02-01 |  | 0.228804 |  |
| 2018-03-01 |  | 0.224865 |  |
| 2018-04-01 |  | 0.233389 |  |
| 2018-05-01 |  | 0.257624 |  |
| 2018-06-01 |  | 0.227047 |  |
| 2018-07-01 |  | 0.17781 |  |
| 2018-08-01 |  | 0.21358 |  |
| 2018-09-01 |  | 0.227518 |  |
| 2018-10-01 |  | 0.23017 |  |
| 2018-11-01 |  | 0.22997 |  |
| 2018-12-01 |  | 0.280655 |  |
| 2019-01-01 |  | 0.266292 |  |
| 2019-02-01 |  | 0.24118 |  |
| 2019-03-01 |  | 0.222573 |  |
| 2019-04-01 |  | 0.221028 |  |
| 2019-05-01 |  | 0.206758 |  |
| 2019-06-01 |  | 0.200059 |  |
| 2019-07-01 |  | 0.197765 |  |
| 2019-08-01 |  | 0.205646 |  |
| 2019-09-01 |  | 0.216938 |  |
| 2019-10-01 |  | 0.207544 |  |
| 2019-11-01 |  | 0.21142 |  |
| 2019-12-01 |  | 0.222721 |  |
| 2020-01-01 |  | 0.238664 |  |
| 2020-02-01 |  | 0.240277 |  |
| 2020-03-01 |  | 0.271486 |  |
| 2020-04-01 |  | 0.396782 |  |
| 2020-05-01 |  | 0.279961 |  |
| 2020-06-01 |  | 0.227786 |  |
| 2020-07-01 |  | 0.202259 |  |
| 2020-08-01 |  | 0.220839 |  |
| 2020-09-01 |  | 0.225459 |  |
| 2020-10-01 |  | 0.238375 |  |
| 2020-11-01 |  | 0.266206 |  |
| 2020-12-01 |  | 0.312576 | 0.212847 |
| 2021-01-01 |  | 0.296102 |  |
| 2021-02-01 |  | 0.287668 |  |
| 2021-03-01 |  | 0.257655 |  |
| 2021-04-01 |  | 0.218498 |  |
| 2021-05-01 |  | 0.208109 |  |
| 2021-06-01 |  | 0.189562 |  |
| 2021-07-01 |  | 0.165561 |  |
| 2021-08-01 |  | 0.188817 |  |

^a^Source: Iowa State University, https://www.extension.iastate.edu/agdm/energy.html, accessed July 27, 2025.

**Figure B2. Average revenue shares of SBM in soybean processing products, 2007-2024^a^**

|  | weekly share | 5-yr moving avg |
| --- | --- | --- |
| 2007-08-01 | 0.578537 |  |
| 2007-09-01 | 0.576502 |  |
| 2007-10-01 | 0.564531 |  |
| 2007-11-01 | 0.580202 |  |
| 2007-12-01 | 0.566922 |  |
| 2008-01-01 | 0.545908 |  |
| 2008-02-01 | 0.529413 |  |
| 2008-03-01 | 0.534028 |  |
| 2008-04-01 | 0.523761 |  |
| 2008-05-01 | 0.549301 |  |
| 2008-06-01 | 0.571226 |  |
| 2008-07-01 | 0.575515 |  |
| 2008-09-01 | 0.603033 |  |
| 2008-10-01 | 0.593204 |  |
| 2008-11-01 | 0.631612 |  |
| 2008-12-01 | 0.647974 |  |
| 2009-01-01 | 0.659367 |  |
| 2009-02-01 | 0.671222 |  |
| 2009-03-01 | 0.672787 |  |
| 2009-04-01 | 0.661675 |  |
| 2009-05-01 | 0.674312 |  |
| 2009-06-01 | 0.695986 |  |
| 2009-07-01 | 0.7015 |  |
| 2009-08-01 | 0.702063 |  |
| 2009-09-01 | 0.705051 |  |
| 2009-10-02 | 0.662919 |  |
| 2009-11-02 | 0.645912 |  |
| 2009-12-03 | 0.648338 |  |
| 2010-01-03 | 0.646327 |  |
| 2010-02-03 | 0.636096 |  |
| 2010-03-06 | 0.607254 |  |
| 2010-04-06 | 0.611648 |  |
| 2010-05-07 | 0.620604 |  |
| 2010-06-07 | 0.640454 |  |
| 2010-07-08 | 0.650901 |  |
| 2010-08-08 | 0.641066 |  |
| 2010-09-01 | 0.615708 |  |
| 2010-10-02 | 0.594543 |  |
| 2010-11-02 | 0.591483 |  |
| 2010-12-03 | 0.581412 |  |
| 2011-01-03 | 0.575631 |  |
| 2011-02-03 | 0.568932 |  |
| 2011-03-06 | 0.559634 |  |
| 2011-04-06 | 0.54067 |  |
| 2011-05-07 | 0.549364 |  |
| 2011-06-07 | 0.553882 |  |
| 2011-07-08 | 0.555108 |  |
| 2011-08-08 | 0.562427 | 0.607832 |
| 2015-09-01 | 0.715921 |  |
| 2015-10-02 | 0.704578 |  |
| 2015-11-02 | 0.698337 |  |
| 2015-12-03 | 0.6563 |  |
| 2016-01-03 | 0.653778 |  |
| 2016-02-03 | 0.642979 |  |
| 2016-03-05 | 0.634868 |  |
| 2016-04-05 | 0.645154 |  |
| 2016-05-06 | 0.702438 |  |
| 2016-06-06 | 0.722969 |  |
| 2016-07-07 | 0.714403 |  |
| 2016-08-07 | 0.678825 |  |
| 2016-09-01 | 0.670225 |  |
| 2016-10-02 | 0.648688 |  |
| 2016-11-02 | 0.645782 |  |
| 2016-12-03 | 0.636719 |  |
| 2017-01-03 | 0.659981 |  |
| 2017-02-03 | 0.670953 |  |
| 2017-03-06 | 0.667973 |  |
| 2017-04-06 | 0.667798 |  |
| 2017-05-07 | 0.661477 |  |
| 2017-06-07 | 0.653381 |  |
| 2017-07-08 | 0.65736 |  |
| 2017-08-08 | 0.636991 |  |
| 2017-09-01 | 0.639876 |  |
| 2017-10-02 | 0.656182 |  |
| 2017-11-02 | 0.650812 |  |
| 2017-12-03 | 0.662995 |  |
| 2018-01-03 | 0.669946 |  |
| 2018-02-03 | 0.701544 |  |
| 2018-03-06 | 0.713517 |  |
| 2018-04-06 | 0.720451 |  |
| 2018-05-07 | 0.727654 |  |
| 2018-06-07 | 0.714759 |  |
| 2018-07-08 | 0.714116 |  |
| 2018-08-08 | 0.705675 |  |
| 2018-09-01 | 0.695055 |  |
| 2018-10-02 | 0.682523 |  |
| 2018-11-02 | 0.688104 |  |
| 2018-12-03 | 0.683217 |  |
| 2019-01-03 | 0.683295 |  |
| 2019-02-03 | 0.669022 |  |
| 2019-03-06 | 0.674065 |  |
| 2019-04-06 | 0.679999 |  |
| 2019-05-07 | 0.684101 |  |
| 2019-06-07 | 0.692298 |  |
| 2019-07-08 | 0.683381 |  |
| 2019-08-08 | 0.670039 | 0.677302 |
| 2019-09-08 | 0.663742 |  |
| 2019-10-09 | 0.669126 |  |
| 2019-11-09 | 0.665338 |  |
| 2019-12-10 | 0.651241 |  |
| 2020-01-10 | 0.644863 |  |
| 2020-02-10 | 0.660818 |  |
| 2020-03-12 | 0.695055 |  |
| 2020-04-12 | 0.696015 |  |
| 2020-05-13 | 0.696936 |  |
| 2020-06-13 | 0.683121 |  |
| 2020-07-14 | 0.667531 |  |
| 2020-08-14 | 0.639368 | 0.67444 |
| 2020-09-14 | 0.64682 |  |
| 2020-10-15 | 0.680273 |  |
| 2020-11-15 | 0.671674 |  |
| 2020-12-16 | 0.657636 |  |
| 2021-01-16 | 0.659824 |  |
| 2021-02-16 | 0.633952 |  |
| 2021-03-19 | 0.59574 |  |
| 2021-04-19 | 0.562642 |  |
| 2021-05-20 | 0.522861 |  |
| 2021-06-20 | 0.495251 |  |
| 2021-07-21 | 0.492695 |  |
| 2021-08-21 | 0.49769 | 0.658601 |
| 2021-09-21 | 0.501033 |  |
| 2021-10-22 | 0.466759 |  |
| 2021-11-22 | 0.509302 |  |
| 2021-12-23 | 0.545735 |  |
| 2022-01-23 | 0.550699 |  |
| 2022-02-23 | 0.552519 |  |
| 2022-03-26 | 0.553353 |  |
| 2022-04-26 | 0.520121 |  |
| 2022-05-27 | 0.490658 |  |
| 2022-06-27 | 0.515551 |  |
| 2022-07-28 | 0.566737 |  |
| 2022-08-28 | 0.573361 | 0.618357 |
|  | 0.561193 |  |
| 2022-09-28 | 0.553016 |  |
| 2022-10-29 | 0.522516 |  |
| 2022-11-29 | 0.571728 |  |
| 2022-12-30 | 0.588894 |  |
| 2023-01-30 | 0.606783 |  |
| 2023-03-02 | 0.614636 |  |
| 2023-04-02 | 0.61119 |  |
| 2023-05-03 | 0.608679 |  |
| 2023-06-03 | 0.570741 |  |
| 2023-07-04 | 0.547128 |  |
| 2023-08-04 | 0.543826 | 0.591591 |
| 2023-09-04 | 0.545952 |  |
| 2023-10-05 | 0.585765 |  |
| 2023-11-05 | 0.628054 |  |
| 2023-12-06 | 0.621012 |  |
| 2024-01-06 | 0.598503 |  |
| 2024-02-06 | 0.595017 |  |
| 2024-03-08 | 0.597221 |  |
| 2024-04-08 | 0.602589 |  |
| 2024-05-09 | 0.630323 |  |
| 2024-06-09 | 0.631652 |  |
| 2024-07-10 | 0.602506 |  |

^a^Source: Oil Crops Yearbook ((Economic Research Service (ERS) 2025)), <https://www.ers.usda.gov/data-products/oil-crops-yearbook>, accessed July 27, 2025. Gaps in data 2011-2015 occurred in the report.
